# Involvement of a coumarin analog AD-013 in the DNA damage response pathways in MCF-7 cells

**DOI:** 10.1007/s11033-018-4271-z

**Published:** 2018-08-07

**Authors:** Angelika Długosz, Joanna Drogosz, Dariusz Deredas, Tomasz Janecki, Anna Janecka

**Affiliations:** 1 Department of Biomolecular Chemistry, Faculty of Medicine, Medical University of Lodz, Mazowiecka 6/8, 92-215 Lodz, Poland; 2 Institute of Organic Chemistry, Faculty of Chemistry, Lodz University of Technology, Lodz, Poland

**Keywords:** DNA damage, ATM, BRCA1, P53, Apoptosis

## Abstract

Coumarin is a plant-derived compound but as such has no medical uses. Several synthetic coumarin analogs have been shown to possess anti-proliferative activity and to induce apoptosis in cancer cells. Here, we explored DNA damage responses in MCF-7 cells treated with our novel synthetic hybrid compound AD-013, which integrates a coumarin moiety and an α-methylene-δ-lactone motif. The mRNA expression of several genes engaged in DNA-damage-induced responses was assessed by quantitative real-time PCR. The protein levels of a few members of phosphoinositide-3-kinases family (ATM, ATR and DNA-PK) and BRCA1 were assessed by ELISA, while p53 was evaluated by western blot method. AD-013 down-regulated *DNA-PK* gene expression but increased the level of ATM/ATR and p53. The new analog completely inhibited *BRCA1* and greatly decreased the activity of BRCA1 protein, engaged in DNA damage repair. Exposure of MCF-7 cells to a coumarin analog AD-013 led to DNA damage and decreased expression of several repair-associated genes.

## Introduction

Coumarin is a natural phytochemical but as such has no medical uses. However, the motif of coumarin is present in many natural compounds with promising therapeutic properties. In the last few years, coumarin analogs have attracted considerable interest, since some of them were shown to possess anti-proliferative activity and to induce apoptosis in cancer cells [1, 2].

**Fig. 1:**
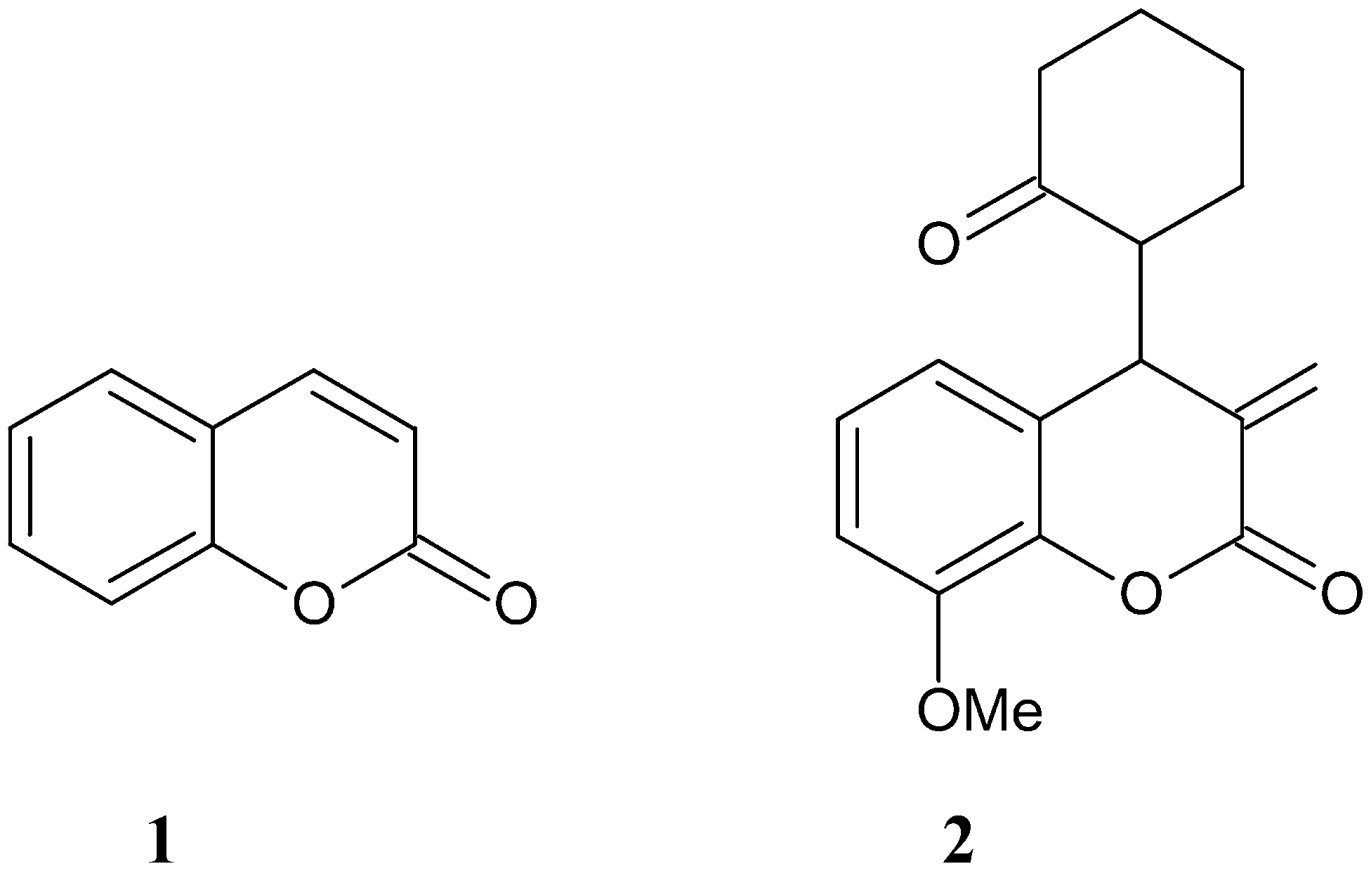
Structure of coumarin (**1**) and its analog (R*)-8-methoxy-3-methylene-4-[(S*)-2-oxocyclohexyl]chroman-2-one, designated AD-013 (**2**)

Recently, we have published a new coumarin analog, (R*)-8-methoxy-3-methylene-4-[(S*)-2-oxocyclohexyl]chroman-2-one, designated AD-013, which combines a coumarin framework with an α-methylene-δ-lactone motif (Fig. 1) [3].

AD-013 was tested against MCF-7 and HL-60 cancer cell lines. Since the cytotoxic activity of this analog was higher in MCF-7 cells, further experiments were performed using this cell line. AD-013 showed the pro-apoptotic activity, significantly increasing expression levels of the pro-apoptotic genes (*Bax, caspase-9, caspase-3*) and down-regulating anti-apoptotic genes (*Bcl-2* and *Bcl-xl*) and also up-regulated the mRNA level of *p53*. All these events led to the significant increase in the number of apoptotic cells. On the other hand, expression of cyclins (*CCNE1* and *CCND1*) and cyclin-dependent kinase 2 (*CDK-2*) was up-regulated. The CDK2/E cyclin complex is responsible for the progression of cells from G1 to S phase. AD-013 decreased the mRNA level of *p21*, an inhibitor of CDK2 activity, indicating that cell cycle was not arrested in G1/S. It was also shown that AD-013 was able to induce DNA damage in almost 80% and inhibit MCF-7 proliferation in about 96% of cell population [3].

The aim of this study was to investigate DNA damage responses (DDR) in MCF-7 cells treated with AD-013.

To avoid death, cells (including cancer cells) have evolved a complex network of DDR systems [4]. DDR is a collective term for various events triggered by aberrant DNA. In general, this pathway consists of hierarchically arranged signal sensors-proteins detecting DNA damage and activating kinases, including transducers and mediator proteins facilitating phosphorylation of effectors [5–7]. Effectors in turn evoke cell-cycle arrest, activation of DNA repair mechanisms or programmed cell death (apoptosis) when repair is not possible [6, 8–10].

Generally, DNA damage may be linked to activation of phosphoinositide-3-kinases family (PI3Ks) [11] which includes ataxia-teleangiectasia-mutated (ATM) proteins, ataxia telangiectasia and Rad3-related (ATR) proteins and DNA-dependent protein kinase catalytic subunit (DNA-PKcs) [12, 13]. These proteins are primary mediators of the response to double-strand breaks (DSBs) in DNA and may control the cell cycle by phosphorylation of checkpoint 1 or checkpoint 2 protein kinases (Chk1 or Chk2), therefore functioning as regulators of the cell cycle at the G1/S or G2/M phases [14].

In mammalian cells, ATM and ATR phosphorylate the Ser/Thr-Glu motifs and ATM- or ATR-dependent sites in many cellular proteins, while DNA-PK regulates a small number of effectors engaged only in the non-homologous end joining pathway (NHEJ) [15–19].

Activation of ATM protein kinase is also required for phosphorylation of tumor suppressor, known as breast cancer gene 1 (*BRCA1*). The over-expression of this gene is important in cellular pathways that maintain genomic stability, such as DNA damage-induced cell cycle checkpoint activation, DNA repair and apoptosis [20–23].

Phosphoinositide-3-kinases family also participate in regulation of p53 protein activity [24, 25] directly by phosphorylation of Ser15 or indirectly through activation of Chk2 or Chk1 [26]. Phosphorylated p53 plays a significant role in modulation of the cell cycle by induction the G1-, S-, or G2 phase arrest or stimulation of apoptosis when DNA is damaged [26–28].

A connection between transcription, cell cycle arrest and DNA damage was demonstrated through the assessment of TATA box-binding protein (TBP). As well known, the specific TATA box factor is involved in principal mechanisms of transcription by combining with the gene promoter. Moreover, TBP protein can regulate the activity of p53 protein, checkpoints, proliferation and apoptosis through potentiation of gene expression [29, 30].

Cell differentiation, division and stress response are also connected with activity of *ABL-1* protooncogene. Interestingly, ABL-1-deficient cells failed to exhibit effective DNA damage-induced phosphorylation of p53 executed by ATM, ATR or DNA-PK [31].

When the level of damage is not severe, cell cycle checkpoints are activated which results in enhancement of DNA repair pathways. However, excessive DNA damage leads to initiation of apoptosis.

Here, we investigated the influence of AD-013 on DNA-damage response pathways in MCF-7 cell line.

## Materials and methods

### Materials

A synthetic analog, (R*)-8-methoxy-3-methylene-4-[(S*)-2-oxocyclohexyl]chroman-2-one (AD-013) was obtained in a two-step reaction sequence published elsewhere [32]. For all experiments the tested compound was dissolved in dimethyl sulfoxide (DMSO) and then diluted in an appropriate culture medium to obtain the final concentration of DMSO less than 0.1% v/v. In each test controls without and with 0.1% DMSO were performed.

### Cell culture

Breast cancer MCF-7 cell line was obtained from the European Collection of Cell Cultures (ECACC). MCF-7 cells were maintained in EMEM growth medium, supplemented with 10% fetal bovine serum (FBS), 1% NEAA, 2 mM glutamine and antibiotics, in an atmosphere containing 5% CO_2_ in humidified air at 37 °C.

### MTT cell proliferation/viability assay

The effect of AD-013 on the viability and proliferation of MCF-7 cells was determined by 3-(4,5-dimethylthiazol-2-yl)-2,5 diphenyl tetrazolium bromide (MTT) assay, according to the Mosmann method, as described elsewhere [32, 33].

### Quantitative real-time PCR assay

The mRNA levels of genes involved in DNA-damage-induced responses were assessed using quantitative real-time PCR.

Briefly, MCF-7 cells seeded on 6-well plates (4.5 × 10^5^ cells/well) were incubated for 24 h with AD-013 at IC_50_ concentration. The cells cultured without the tested analog were used as control. Then, cells were washed twice with PBS, detached and collected by centrifugation (200×*g*, 5 min). Total RNA was extracted using Total RNA Mini Kit (A&A Biotechnology, Poland), combining the standard TRIzol and column*-*based methods, while cDNA was synthesized using Transcriba Kit (A&A Biotechnology, Poland), both according to the manufacturer’s protocol.

The concentration of RNA was measured using sensitive single-tube fluorimeter for fluorescence-based quantitation of nucleic acids and proteins. The obtained value was 90 ng/µl.

cDNA was amplified using the pre-designed 96-well panel with immobilized gene specific primers (*ATM, ATR, ABL-1, BRCA1, CHEK1, CHEK2,PRKDC, TBP, TP53*) (Bio-rad, United Kingdom) and sensitive Real-Time 2xHS-PCR SYBR Master Mix (A&A Biotechnology, Poland) in Stratagene MX3005P QPCR System (Agilent Technologies, Inc. Santa Clara, CA, USA). Real-time PCR was performed in a 20 µl reaction volume according to the manufacturer’s instructions. Glyceraldehyde 3-phosphate dehydrogenase (*GAPDH*) was used as a reference gene.

Real-time PCR cycles were run using the following thermal cycling profile: initial denaturation at 95 °C for 2 min and 40 cycles of denaturation at 95 °C for 5 s, annealing at 60 °C for 30 s and extension at 72 °C for 30 s. The expression levels of the tested genes were determined by the 2^−∆∆CT^ method [34].

### Evaluation of phospho- and total-ATM activity by ELISA-based method

ATM activity was analyzed in protein extracts (20 µg) by the ELISA-based method using RayBio Phospho-ATM (Ser1981) and Total ATM protein kit (RayBiotech, Norcross, GA, USA).

Cells seeded on 6-well plates (4.5 × 10^5^ cells/well) were incubated with AD-013 at IC_50_ or 2×IC_50_ concentration, for 24 h. Then, cells were washed with PBS and collected by centrifugation (200×*g*, 5 min). Nuclear extracts were prepared using the buffer for cell lysis. Then, extracts were analyzed using anti-pan ATM and anti-phospho-ATM (Ser1981) antibodies coated onto a 96-well plate. ATM proteins present in a sample specifically bind to the wells by these immobilized antibodies. For detection of the ATM activity, secondary antibodies, HRP-conjugated anti-rabbit IgG and HRP-Streptavidin, were used. Addition of TMB (3,3,5,5′-tetramethylbenzidine) substrate solution and stop solution provided sensitive colorimetric readout, easily quantified spectrophotometrically.

### Human BRCA1 (breast cancer susceptibility protein 1) ELISA kit

Determination of BRCA1 protein level in MCF-7 cells incubated with AD-013 was analyzed by Human BRCA1 ELISA kit (Elabscience, Houston, TX, USA).

MCF-7 cells seeded on 6-well plates (5.0 × 10^4^ cells/well) were incubated for 24 h with the tested compound at IC_50_ concentration. Then, cells washed with PBS were collected by centrifugation (200×*g*, 5 min). Nuclear extract was prepared according to the manufacturer’s instructions. Each extract was analyzed using an ELISA-based method.

The micro 96-well plate was pre-coated with an antibody specific to BRCA1. Standards were added to the micro ELISA plate and combined with the specific antibody. BRCA1 protein present in a sample was specifically bound to the wells by immobilized antibodies. Then, detectible secondary antibodies conjugated with horseradish peroxidase (HRP) bound the primary antibodies (the color turned blue) that provided sensitive colorimetric readout. The substrate-enzyme reaction was terminated by the addition of stop solution (the color turned yellow). The optical density (OD) was measured spectrophotometrically at a wavelength of 450 nm.

### Western blot

The determination of p53 protein level was performed by Western blot analysis.

Briefly, MCF-7 cancer cells seeded on 6-well plates (5 × 10^5^ cells/well) were incubated for 24 h with AD-013 at IC_50_ concentration. The cells cultured without the tested compound were used as control, while Jurkat cell lysates as a positive control. After incubation, cells were lysed in a RIPA buffer (containing 150 mM NaCl, 1.0% IGEPAL® CA-630, 0.5% sodium deoxycholate, 0.1% SDS, 50 mM Tris, pH 8.0.), collected by centrifugation (200×*g*, 5 min) and the pellets were removed. Then, 20 µg of total protein extract from supernatant was separated on SDS-PAGE gel and transferred onto nitrocellulose blotting membranes (Amershan, Germany) according to the manufacturer’s instructions. After separation, western blotting membrane was blocked with 5% skim milk and then incubated with primary [anti-p53(E26) (Abcam, concentration 1:1000), anti-beta Actin (Abcam, concentration 1:1000)] and secondary [goat anti-rabbit IgG H&L (HRP) (Abcam, concentration 1:50,000)] antibodies at room temperature for 1.5 h each. Protein bands were visualized by SuperSignal west pico chemiluminescent substrate (Thermo Scientific, Waltham, MA, USA).

### Statistical analysis

Results were expressed as mean ± SEM, using Prism 5.0 (GraphPad Software Inc., San Diego, CA, USA). Statistical significance was analyzed by one-way ANOVA followed by a post-hoc multiple comparison Student-Newman-Keuls test or Student *t* test, *p < 0.05, **p < 0.01, and ***p < 0.001 was considered statistically significant.

## Results

### Cell viability assay (MTT)

**Fig. 2:**
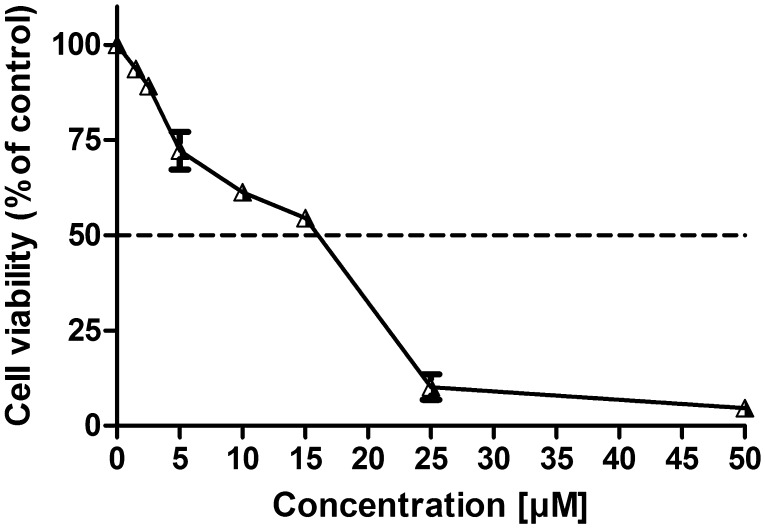
The cytotoxic effect of AD-013 on MCF-7 cells analyzed by MTT assay

The effect of a novel coumarin analog AD-013 on MCF-7 cell growth was assessed using the MTT assay. After 24 h exposure of the cells to a broad range of compound concentrations AD-013 showed high, dose-dependent cytotoxic activity (Fig. 2). The IC_50_ value, which represents concentration of a compound required to inhibit metabolic activity of 50% of cells, was 16.76 ± 0.25 µM. This concentration was used in all further experiments.

### Effect of AD-013 on expression levels of selected genes involved in DNA damage

The changes in mRNA expression of *ATM, ATR, BRCA1, CHEK1, CHEK2, PRKDC, TBP, TP53* and *ABL-1* genes in MCF-7 cells treated for 24 h with AD-013 (at IC_50_ concentration) were assessed by real-time PCR. Gene expression was normalized to the house keeping *GAPDH* gene.

**Fig. 3:**
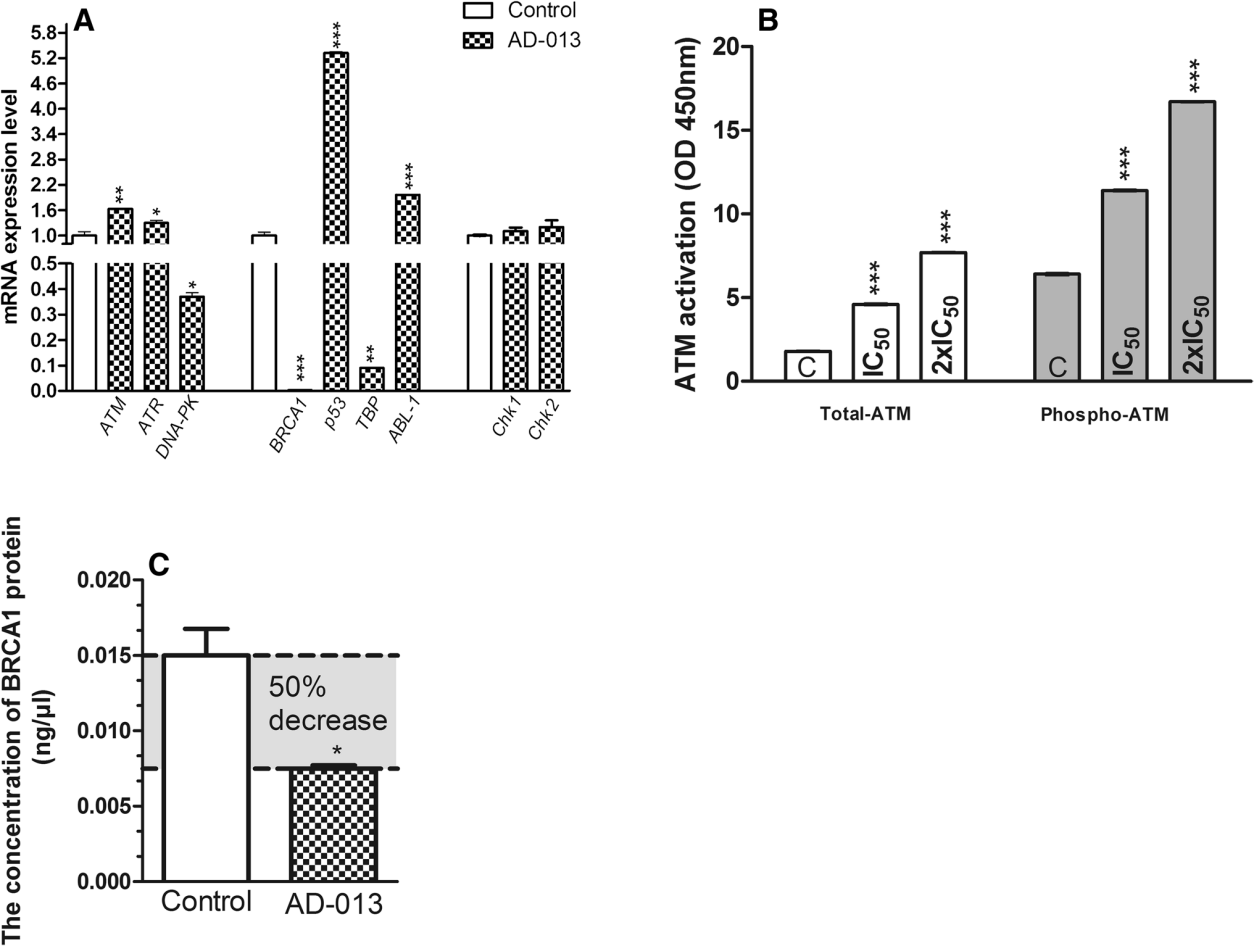
**A** Real-time PCR analysis of ATM, ATR, DNA-PK, BRCA1, p53, TBP, ABL-1, Chk1 and Chk2 mRNA levels in MCF-7 cells treated with AD-013. Data are expressed as mean ± SEM. Statistical significance was assessed by the Student *t*-test. *p < 0.05 and **p < 0.01 was considered as significantly different from control. **B** Effect of AD-013 on phosphorylation of ATM Ser1981 in MCF-7 cells. Protein extracts obtained after treatment of cells with the tested compound (at IC_50_ and 2×IC_50_ concentrations) were assayed for ATM phosphorylation (activation), using the RayBio Phospho-ATM (Ser1981) and Total ATM protein Kit. Data are expressed as mean ± SEM. Statistical significance was assessed using one-way ANOVA and a post-hoc multiple comparison Student–Newman–Keuls test. ***p < 0.001, in comparison with control. **C** Influence of AD-013 on BRCA1 protein concentration in MCF-7 cells. Extracts from MCF-7 cells treated with AD-013 at IC_50_ concentration were assayed for BRCA1 activation, using the Human BRCA1 ELISA kit. Data are presented as mean ± SEM. Statistical significance was assessed by the Student *t* test *p < 0.05, in comparison with control

Our analyses revealed that AD-013 increased the mRNA level of *ATM* and *ATR* but significantly down-regulated the expression of *DNA-PK* in MCF-7 cells, as compared with control (Fig. 3A). The tested compound did not influence *Chk1* and *Chk2* expression (Fig. 3A).

As demonstrated in Fig. 3A, AD-013 significantly decreased the mRNA level of *BRCA1* in MCF-7 cells. The obtained values were close to zero. Additionally, AD-013 caused up-regulation of *p53* and *ABL-1* expression and slightly decreased mRNA level of *TBP*.

### Analysis of ATM activity by ELISA-based method

RayBio Phospho-ATM (Ser1981) and Total ATM protein kit was used to quantify the level of ATM in cancer cell lysates. Phosphorylation of ATM Ser1981 in MCF-7 cells was investigated after 24 h exposure of cells to AD-013 at IC_50_ and 2×IC_50_ concentration.

The obtained results demonstrated that this analog significantly and concentration-dependently increased both, the level of phosphorylated and total-ATM proteins in MCF-7 cells but the up-regulation of phospho-ATM was more pronounced (twofold higher) (Fig. 3B).

### Assessment of BRCA1 protein level in MCF-7

To measure BRCA1 concentration in cancer cell lysates, the Human BRCA1 ELISA kit was used. MCF-7 cancer cell lysates were prepared after 24 h exposure of cells to AD-013 at IC_50_ concentration.

As shown in Fig. 3C, AD-013 decreased by 50% the protein level of BRCA1 in MCF-7 cells.

### Detection of p53 protein level by Western blotting

**Fig. 4:**
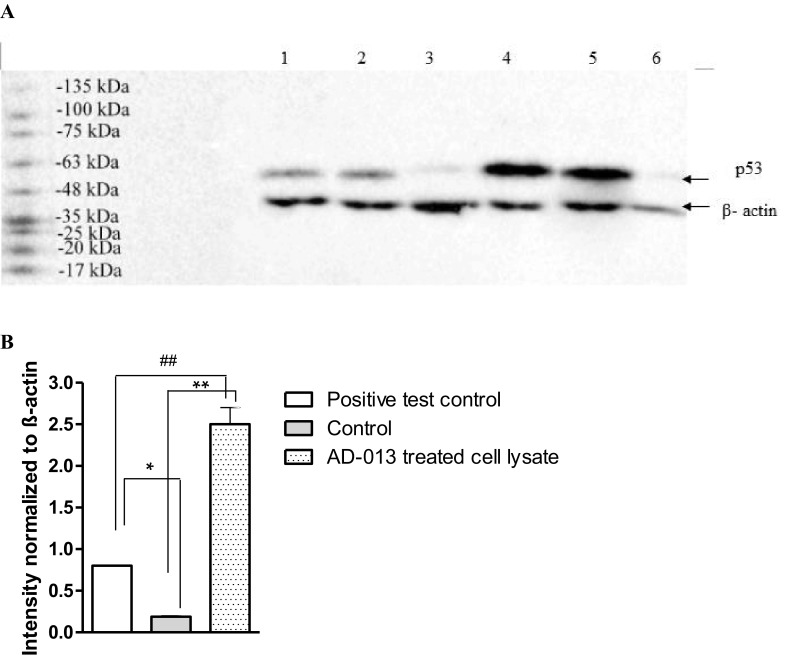
Western blot analysis and quantification of p53 protein level in MCF-7. **A** Representative western blot: 1,2-positive test control (Jurkate cell lysate), 3,6-control (MCF-7 cell lysate), 4,5-AD-013 treated cell lysate. **B** Densitometry analysis. The level of p53 expression was normalized to β-actin. Using one-way ANOVA and a post-hoc multiple comparison Student–Newman–Keuls test **p < 0.01, *p < 0.05, in comparison with control; ^##^*p* < 0.01

To assess the p53 protein level in MCF-7 cells, Western blotting analysis was performed using appropriate antibodies. Lysates were prepared after 24 h exposure of the cells to AD-013 at IC_50_ concentration. In obtained protein extracts, relatively high level of 53 kDa p53 protein was observed 12.5-fold increase as compared with control (MCF-7 cell lysate) (Fig. 4).

## Discussion

Understanding the molecular basis of DNA-damage and DDR pathways is important not only for better comprehension of carcinogenesis, but also in the development of effective anti-cancer drugs.

ATM, ATR and DNA-PK are three related kinases controlling DDR pathway [5]. DNA-PKcs is activated when DSBs occur and its major role is to promote non-homologous end joining (NHEJ). NHEJ efficiently repairs most DSBs by ligation of two broken DNA ends [6, 35]. AD-013 significantly decreased the level of *DNA-PK*, therefore preventing DNA repair by NHEJ pathway.

The most upstream DDR regulators, ATM and ATR proteins, in response to DNA breaks, undergo a rapid increase in their kinase activity [36–38]. The obtained data indicated that AD-013 up-regulated in MCF-7 cells the expression of both, *ATM* and *ATR*, but more significantly *ATM*. The levels of phosphorylated and total-ATM proteins were also increased.

Chk1, Chk2 and p53 protein are crucial phosphorylation targets of ATM and ATR. In response to the DNA damage, ATM and ATR may phosphorylate Chk2 and Chk1, respectively. Both Chk2 and Chk1 also phosphorylate and stabilize p53 protein. However, p53 can be directly phosphorylated by ATM or ATR, resulting in the increase of its transcriptional activity [39]. In our study AD-013 did not change expression levels of *Chk2* and *Chk1* but significantly increased gene and protein level of p53. That caused *p53* target genes, such as pro- and anti-apoptotic genes, to be transcriptionally induced, as was shown in our previous study [3].

The transcriptional activity of *p53* can be also regulated by *TBP* and *ABL-1* genes. Here, the up-regulation of *ABL-1* and down-regulation of *TBP* suggested that p53 had to be directly phosphorylated by ATM.

Another important element in the DDR pathway is tumor suppressor gene *BRCA1*. Phosphorylation of BRCA1 occurs during S phase of the cell cycle but also in response to DNA damage [40]. BRCA1 has been suggested to be responsible for activation of all checkpoints (G1/S, S-phase and G2/M) [41]. This protein is also important in cellular pathways that maintain genomic stability, including DNA damage-induced cell cycle checkpoint activation, DNA repair and apoptosis [20–23]. In MCF-7 cells exposed to AD-013, the *BRCA1* gene and BRCA1 protein expression were very low, close to zero. Therefore, BRCA1 deficiency could lead to the lack of checkpoint activation and defects of DNA repair pathway.

ATM and ATR phosphorylate their numerous substrates and thus induce DDR [36–38]. Phosphorylation of substrates of ATM, such as BRCA1, Chk2 and p53, may lead to different downstream processes including DNA repair, cell-cycle arrest or apoptosis [6, 10, 40].

**Fig. 5:**
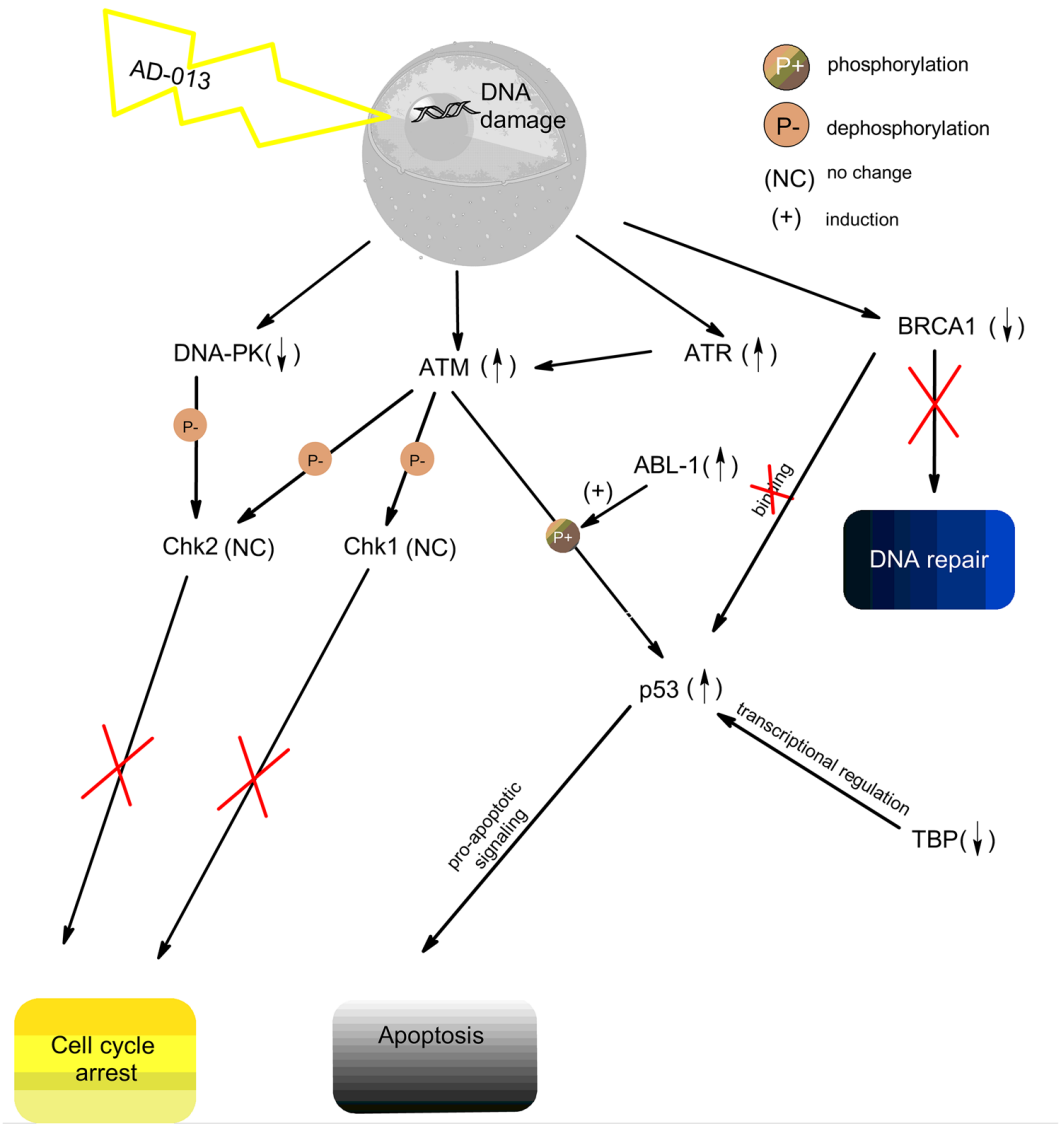
The possible response mechanisms to DNA damage induced by AD-013 in MCF-7 cells

The graph in Fig. 5 shows that AD-013 induced in MCF-7 cells DNA damage, followed by the activation of ATM, ATR and p53 and down-regulation of DNA repair-associated genes *BRCA1* and *DNA-PK*, resulting in the DNA repair defects and apoptosis.

## Conclusions

Repair mechanisms of cancer cells in response to DNA damage caused by anticancer drugs greatly affect the efficacy of such treatments. Understanding these mechanisms is essential in the search for new potential anticancer agents.

In this communication we investigated DNA damage responses in MCF-7 cells, following exposure to AD-013, our previously published synthetic coumarin analog.

The obtained results proved that inhibition of DNA-PK and BRCA1 activity resulted in the defects of DNA repair pathway while induction of ATM/ATR and p53 led to apoptosis.

The data presented here indicate that AD-013, which combines a coumarin scaffold with an α-methylene-δ-lactone motif, shows potential in the search for new chemotherapeutic agents against breast cancer.

## Abbreviations

ABL-1: Abelson murine leukemia viral oncogene homolog 1
ATCC: American type culture collection
ATM: Ataxia-teleangiectasia-mutated protein
ATR: Ataxia telangiectasia and Rad3-related protein
BRCA1: Breast cancer gene 1
CCND1: Cyclin D1
CCNE1: Cyclin E1
CDK-2: Cyclin-dependent kinase 2
Chk1: Checkpoint 1 protein kinase
Chk2: Checkpoint 2 protein kinase
DDR: DNA damage responses
DNA: PKcs-DNA-dependent protein kinase catalytic subunit
DSB: Double-strand break
ECACC: European collection of cell cultures
FBS: Fetal bovine serum
HRP: Horseradish peroxidase
IC_50_: The concentration of a drug that is required for 50% inhibition in vitro
MTT: 3-(4,5-Dimethylthiazol-2-yl)-2,5 diphenyl tetrazolium bromide
NEAA: Non-essential amino AIDS
NHEJ: Non-homologous end joining pathway
PI3K: Phosphoinositide-3-kinases family
RIPA: Radioimmunoprecipitation assai
SSB: Single-strand break
TBP: TATA box-binding protein

## References

[1] Riveiro ME, Vazquez R, Moglioni A, Gomez NA (2008). Biochemical mechanisms underlying the pro-apoptotic activity of 7,8-dihydroxy-4-methylcoumarin in human leukemic cells. Biochem Pharmacol.

[2] Zhang L, Jiang G, Yao F, He Y, Liang G, Zhang Y, Hu B, Yan W, Li Y, Liu H (2012). Growth inhibition and apoptosis induced by osthole, a natural coumarin, in hepatocellular carcinoma. PLoS ONE.

[3] Długosz A, Gach-Janczak K, Szymański J, Deredas D, Krawczyk H, Janecki T, Janecka A (2017). Anticancer properties of a new hybrid analog AD-013 combining a coumarin scaffold with an α-methylene-δ-lactone motif.. Anticancer Agents Med Chem.

[4] Giglia-Mari G, Zotter A, Vermeulen W (2011). DNA damage response. Cold Spring Harb Perspect Biol.

[5] Blackford AN, Jackson SP (2017). ATM, ATR, and DNA-PK: The trinity at the heart of the DNA damage response. Mol Cell.

[6] Maréchal A, Zou L (2013). DNA damage sensing by the ATM and ATR kinases. Cold Spring Harb Perspect Biol.

[7] Shiloh Y (2003). ATM and related protein kinases: safeguarding genome integrity. Nat Rev Cancer.

[8] O’Connor MJ (2015). Targeting the DNA damage response in cancer. Mol Cell.

[9] Banin S, Moyal L, Shieh SY, Taya Y, Anderson CW, Chessa L, Smorodinsky NI, Prives C, Reiss Y, Shiloh Y, Ziv Y (1998). Enhanced phosphorylation of p53 by ATM in response to DNA damage. Science.

[10] Roos WP, Kaina B (2013). DNA damage-induced cell death: from specific DNA lesions to the DNA damage response and apoptosis. Cancer Lett.

[11] Broustas CG, Lieberman HB (2014). DNA damage response genes and the development of cancer metastasis. Radiat Res.

[12] Lempiäinen H, Halazonetis TD (2009). Emerging common themes in regulation of PIKKs and PI3Ks. EMBO J.

[13] Lovejoy CA, Cortez D (2009). Common mechanisms of PIKK regulation. DNA Repair.

[14] Manic G, Obrist F, Sistigu A, Vitale I (2015). Trial watch: targeting ATM–CHK2 and ATR–CHK1 pathways for anticancer therapy. Mol Cell Oncol.

[15] Matsuoka S, Ballif BA, Smogorzewska A, McDonald ER, Hurov KE, Luo J, Bakalarski CE, Zhao Z, Solimini N, Lerenthal Y, Shiloh Y, Gygi SP, Elledge SJ (2007). ATM and ATR substrate analysis reveals extensive protein networks responsive to DNA damage. Science.

[16] Smolka MB, Albuquerque CP, Chen SH, Zhou H (2007). Proteome-wide identification of in vivo targets of DNA damage checkpoint kinases. Proc Natl Acad Sci USA.

[17] Stokes MP, Rush J, MacNeill J, Ren JM, Sprott K, Nardone J, Yang V, Beausoleil SA, Gygi SP, Livingstone M, Zhang H, Polakiewicz RD, Comb MJ (2007). Profiling of UV-induced ATM/ATR signaling pathways. Proc Natl Acad Sci USA.

[18] Bensimon A, Schmidt A, Ziv Y, Elkon R, Wang SY, Chen DJ, Aebersold R, Shiloh Y (2010). ATM-dependent and-independent dynamics of the nuclear phosphoproteome after DNA damage. Sci Signal.

[19] Lane SI, Morgan SL, Wu T, Collins JK, Merriman JA, ElInati E, Turner JM, Jones KT (2017). DNA damage induces a kinetochore-based ATM/ATR-independent SAC arrest unique to the first meiotic division in mouse oocytes. Development.

[20] Xiang T, Ohashi A, Huang Y, Pandita TK, Ludwig T, Powell SN, Yang Q (2008). Negative regulation of AKT activation by BRCA1. Cancer Res.

[21] Wang L, Di LJ (2014). BRCA1 and estrogen/estrogen receptor in breast cancer: where they interact?. Int J Biol Sci.

[22] Bouafia A, Corre S, Gilot D, Mouchet N, Prince S, Galibert MD (2014). p53 requires the stress sensor USF1 to direct appropriate cell fate decision. PLoS Genet.

[23] López-Knowles E, O’toole SA, McNeil CM, Millar EK, Qiu MR, Crea P, Daly RJ, Musgrove EA, Sutherland RL (2010). PI3K pathway activation in breast cancer is associated with the basal-like phenotype and cancer-specific mortality. Int J Cancer.

[24] Stavridi ES, Chehab NH, Malikzay A, Halazonetis TD (2001). Substitutions that compromise the ionizing radiation-induced association of p53 with 14-3-3 proteins also compromise the ability of p53 to induce cell cycle arrest. Cancer Res.

[25] Mhawech P (2005). 14-3-3 proteins: an update. Cell Res.

[26] Sancar A, Lindsey-Boltz LA, Ünsal-Kaçmaz K, Linn S (2004). Molecular mechanisms of mammalian DNA repair and the DNA damage checkpoints. Annu Rev Biochem.

[27] Zhu H, Gooderham NJ (2006). Mechanisms of induction of cell cycle arrest and cell death by cryptolepine in human lung adenocarcinoma a549 cells. Toxicol Sci.

[28] Abbas T, Dutta A (2009). p21 in cancer: intricate networks and multiple activities. Nat Rev Cancer.

[29] Johnson SA, Dubeau L, Kawalek M, Dervan A, Schönthal AH, Dang CV, Johnson DL (2003). Increased expression of TATA-binding protein, the central transcription factor, can contribute to oncogenesis. Mol Cell Biol.

[30] Bodzak E, Blough MD, Lee PW, Hill R (2008). p53 Binding to the p21 promoter is dependent on the nature of DNA damage. Cell Cycle.

[31] Meltser V, Ben-Yehoyada M, Shaul Y (2011). c-Abl tyrosine kinase in the DNA damage response: cell death and more. Cell Death Differ.

[32] Deredas D, Huben K, Janecka A, Długosz A, Pomorska DK, Mirowski M, Krajewska U, Janecki T, Krawczyk H (2016). Synthesis and anticancer properties of 3-methylene-4-(2-oxoalkyl)-3,4-dihydrocoumarins. Chem Commun.

[33] Mosmann T (1983). Rapid colorimetric assay for cellular growth and survival: application to proliferation and cytotoxicity assays. J Immunol Methods.

[34] Winer J, Jung CK, Shackel I, Williams PM (1999). Development and validation of real-time quantitative reverse transcriptase-polymerase chain reaction for monitoring gene expression in cardiac myocytes in vitro. Anal Biochem.

[35] Jiang W, Crowe JL, Liu X, Nakajima S, Wang Y, Li C, Lee BJ, Dubois RL, Liu C, Yu X, Lan L, Zha S (2015). Differential phosphorylation of DNA-PKcs regulates the interplay between end-processing and end-ligation during nonhomologous end-joining. Mol Cell.

[36] Awasthi P, Foiani M, Kumar A (2016). ATM and ATR signaling at a glance. J Cell Sci.

[37] Guleria A, Chandna S (2016). ATM kinase: Much more than a DNA damage responsive protein. DNA Repair.

[38] Smith J, Tho LM, Xu N, Gillespie DA (2010). The ATM–Chk2 and ATR–Chk1 pathways in DNA damage signaling and cancer. Adv Cancer Res.

[39] Roos WP, Thomas AD, Kaina B (2016). DNA damage and the balance between survival and death in cancer biology. Nat Rev Cancer.

[40] Cortez D, Wang Y, Qin J, Elledge SJ (1999). Requirement of ATM-dependent phosphorylation of brca1 in the DNA damage response to double-strand breaks. Science.

[41] Wu J, Lu LY, Yu X (2010). The role of BRCA1 in DNA damage response. Protein Cell.

